# A proteomics sample metadata representation for multiomics integration and big data analysis

**DOI:** 10.1038/s41467-021-26111-3

**Published:** 2021-10-06

**Authors:** Chengxin Dai, Anja Füllgrabe, Julianus Pfeuffer, Elizaveta M. Solovyeva, Jingwen Deng, Pablo Moreno, Selvakumar Kamatchinathan, Deepti Jaiswal Kundu, Nancy George, Silvie Fexova, Björn Grüning, Melanie Christine Föll, Johannes Griss, Marc Vaudel, Enrique Audain, Marie Locard-Paulet, Michael Turewicz, Martin Eisenacher, Julian Uszkoreit, Tim Van Den Bossche, Veit Schwämmle, Henry Webel, Stefan Schulze, David Bouyssié, Savita Jayaram, Vinay Kumar Duggineni, Patroklos Samaras, Mathias Wilhelm, Meena Choi, Mingxun Wang, Oliver Kohlbacher, Alvis Brazma, Irene Papatheodorou, Nuno Bandeira, Eric W. Deutsch, Juan Antonio Vizcaíno, Mingze Bai, Timo Sachsenberg, Lev I. Levitsky, Yasset Perez-Riverol

**Affiliations:** 1 Chongqing Key Laboratory of Big Data for Bio Intelligence, Chongqing University of Posts and Telecommunications, Chongqing, China; 2 European Molecular Biology Laboratory, European Bioinformatics Institute, Wellcome Genome Campus, Hinxton, UK; 3 Algorithmic Bioinformatics, Freie Universität Berlin, Berlin, Germany; 4 Visualization and Data analysis, Zuse Institute Berlin, Berlin, Germany; 5 Moscow Institute of Physics and Technology, Dolgoprudny, Moscow Region Russia; 6 V.L. Talrose Institute for Energy Problems of Chemical Physics, N.N. Semenov Federal Research Center for Chemical Physics, Russian Academy of Sciences, Moscow, Russia; 7 Bioinformatics Group Department of Computer Science, Albert-Ludwigs-University Freiburg, Freiburg, Germany; 8 Institute for Surgical Pathology, Medical Center – University of Freiburg, Faculty of Medicine, University of Freiburg, Freiburg, Germany; 9 Khoury College of Computer Sciences, Northeastern University, Boston, MA USA; 10 Department of Dermatology, Medical University of Vienna, Vienna, Austria; 11 Department of Clinical Sciences, University of Bergen, Bergen, Norway; 12 Department of Congenital Heart Disease and Pediatric Cardiology, Universitätsklinikum Schleswig-Holstein Kiel, Kiel, Germany; 13 Novo Nordisk Foundation Center for Protein Research, University of Copenhagen, Copenhagen, Denmark; 14 Ruhr University Bochum, Medical Faculty, Medizinisches Proteom-Center, 44801 Bochum, Germany; 15 Ruhr University Bochum, Center for Protein Diagnostics (PRODI), Medical Proteome Analysis, 44801 Bochum, Germany; 16 VIB – UGent Center for Medical Biotechnology, VIB, Ghent, Belgium; 17 Department of Biomolecular Medicine, Faculty of Medicine and Health Sciences, Ghent University, Ghent, Belgium; 18 Department of Biochemistry and Molecular Biology, University of Southern Denmark, Campusvej 55, 5230 Odense, Denmark; 19 University of Pennsylvania, Department of Biology, Philadelphia, PA 19104 USA; 20 Institute of Pharmacology and Structural Biology, University of Toulouse, CNRS, UPS, Toulouse, France; 21 nference Labs, Bengaluru, KA 560017 India; 22 Chair of Proteomics and Bioanalytics, Technical University of Munich, Munich, Germany; 23 Department of Microchemistry, Proteomics and Lipidomics, Genentech, South San Francisco, CA USA; 24 Skaggs School of Pharmacy and Pharmaceutical Sciences, University of California San Diego, La Jolla, CA 92093 USA; 25 Department of Computer Science, Applied Bioinformatics, University of Tübingen, Tübingen, 72076 Germany; 26 Institute for Biological and Medical Informatics, University of Tübingen, Tübingen, 72076 Germany; 27 Institute for Translational Bioinformatics, University Hospital Tübingen, 72076 Tübingen, Germany; 28 Center for Computational Mass Spectrometry, Department of Computer Science and Engineering, Skaggs School of Pharmacy and Pharmaceutical Sciences, University of California, San Diego, CA 92093-0404 USA; 29 Institute for Systems Biology, 401 Terry Ave N, Seattle, WA 98109 USA; 30 State Key Laboratory of Proteomics, Beijing Proteome Research Center, National Center for Protein Sciences (Beijing), Beijing Institute of Life Omics, Beijing, 102206 China

**Keywords:** Proteomics, Data publication and archiving, Proteome informatics, Standardization

## Abstract

The amount of public proteomics data is rapidly increasing but there is no standardized format to describe the sample metadata and their relationship with the dataset files in a way that fully supports their understanding or reanalysis. Here we propose to develop the transcriptomics data format MAGE-TAB into a standard representation for proteomics sample metadata. We implement MAGE-TAB-Proteomics in a crowdsourcing project to manually curate over 200 public datasets. We also describe tools and libraries to validate and submit sample metadata-related information to the PRIDE repository. We expect that these developments will improve the reproducibility and facilitate the reanalysis and integration of public proteomics datasets.

## Abstract (web summary)

The number of publicly available proteomics datasets is growing rapidly, but a standardized approach for describing the associated metadata is lacking. Here, the authors propose a format and a software pipeline to present and validate metadata, and integrate them into ProteomeXchange repositories.

## Introduction

The amount of proteomics data in public repositories is growing at an unprecedented rate^1,2^. ProteomeXchange (PX) is a consortium of proteomics resources, including the PRIDE database^2^, PASSEL and PeptideAtlas^3^, MassIVE^4^, jPOST^5,6^, iProX^7^, and Panorama Public^8^. As of July 2021, over 27,000 datasets have been submitted to PX data repositories. PX datasets cover the whole spectrum of protein mass spectrometry (MS) analytical methods and experimental designs, which enable biologists and clinicians to study different aspects of the proteome. In parallel to the generalization of data deposition, reuse of public datasets is becoming increasingly popular. However, thus far, data reuse has largely been limited to benchmarking studies and applications related to peptide and protein identification, with resources such as PeptideAtlas^3^ and GPMDB^9^ systematically reanalyzing data from PX^10^. Recently, new efforts like ProteomicsDB^11^, MassiVE.Quant^4^, and Expression Atlas^12^ have started to include reanalyzed quantitative public datasets to present baseline and differential protein expression. However, the scalability and broad reuse of public quantitative experiments have been limited by the lack of sample metadata annotation, which unambiguously associates the samples included in each dataset with the corresponding data files^13,14^.

Since 2012, PX resources have been capturing a general dataset description, including the dataset title, description, instrument, protein modifications included in the search, and submitters/principal investigators, among other data^1^. The files included in each dataset are, on one hand, the output of the corresponding instrument (e.g., RAW files), and on the other hand, the processed results, which can be represented, e.g., in standard file formats such as mzIdentML^15^ or mzTab^16^. Currently, all PX partners mandate two types of information for each dataset: a general dataset description and the files containing the different required data types. Unfortunately, the experimental design and sample-related information are frequently missing in the datasets or are stored in ad hoc ways and/or formats^1^. Information about the biological samples such as the analyzed organ, tissue, disease, or cell line, and the links between the samples and the corresponding data files are often lacking.

Sample-related metadata and their relationship with the data files are well captured in two widespread file formats called ISA-TAB^17^ and MAGE-TAB (MicroArray Gene Expression Tabular)^18^, which are used in metabolomics and transcriptomics, respectively. As of May 2021, ArrayExpress has stored over 74,000 functional genomics datasets in the MAGE-TAB format^18,19^. In both formats, a tab-delimited file is used to annotate the sample metadata and link the metadata to the corresponding data files. While MAGE-TAB was originally designed for microarray experiments, it has been successfully adapted to high-throughput RNA-sequencing and single-cell RNA-Seq experiments^20^.

Here we introduce an extension and implementation of the MAGE-TAB format for proteomics (MAGE-TAB-Proteomics). The format has been developed in collaboration with the Proteomics Standards Initiative (PSI), the organization in charge of developing open-standard formats in the field^21^. We have also developed general guidelines about what information needs to be encoded in MAGE-TAB to improve the reproducibility and enable the reanalysis of proteomics datasets. In addition, we have crowdsourced the annotation of over 200 existing public datasets according to these guidelines, covering different analytical methods and experimental designs. Finally, we have developed an ecosystem of tools to validate MAGE-TAB-Proteomics files and integrate the metadata in the PRIDE database, the most popular PX resource. The full specification document describing all aspects of MAGE-TAB-Proteomics version 1.0, the current implementations, as well as application examples, is available at the PSI website (https://psidev.info/magetab).

### Repurposing MAGE-TAB for proteomics

MAGE-TAB encodes the sample metadata annotations and the information linking the metadata to the corresponding data files in two different files: the Investigation Description Format (IDF) and the Sample and Data Relationship Format (SDRF). In the following, we describe how we adapted these formats to the specific needs of proteomics.

### Providing study-description information in IDF

The IDF file contains information describing the study, including, e.g., authors/submitters, protocols, and publications (Supplementary Note 1). The IDF format contains a series of key/value pairs, where each key represents a different property. For example, “Experiment Description” should be followed by a free-text description of the experiment (which would be the value). Most of the fields can contain more than one value, so that multiple values (e.g., multiple-analysis software tools) can be defined in a single IDF file. Since 2012, PX dataset descriptions are provided using PX XML (http://proteomecentral.proteomexchange.org/schemas/proteomeXchange-1.4.0.html), an XML file format that captures equivalent information to the ones included in IDF, making both files easily exchangeable (Supplementary Note 1). Therefore, we developed the IDF component of MAGE-TAB based on the existing PX XML format.

### Linking samples to data files with SDRF

**Fig. 1:**
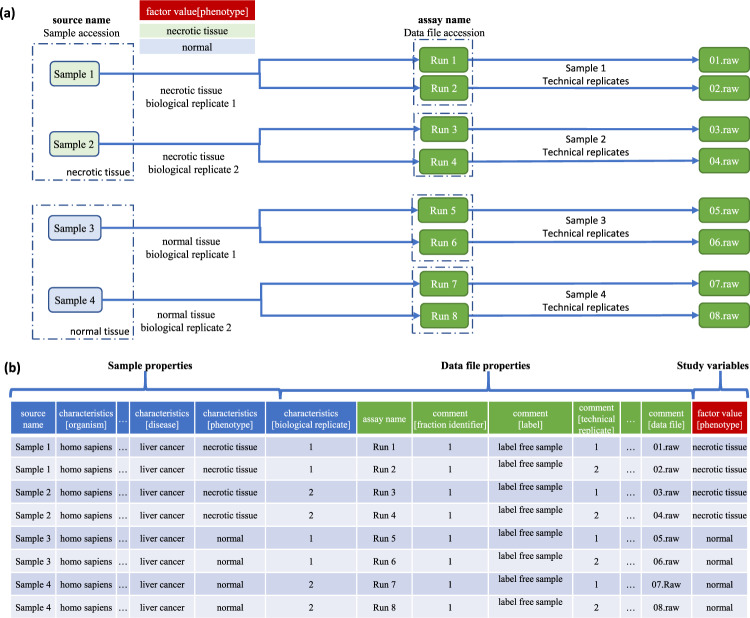
SDRF-Proteomics representation for a label-free-based experiment without fractionation. **a** Experimental design, including two biological replicates and two technical replicates per biological replicate. The biological and technical replicates are defined by the variable under study (e.g., phenotype). **b** The SDRF tab-delimited file, including the three main sections highlighted: sample metadata, data file properties, and the variables under study (factor values).

SDRF is a tab-delimited file that describes the samples and allows their mapping to the data files^1^. As shown in Fig. 1a, SDRF includes the annotation of (i) biological sample metadata; (ii) the relationships between samples and data files; (iii) (technical) metadata of RAW data files; and (iv) the variables under study (called factor values). Each row in an SDRF file corresponds to one relationship between a sample and a data file (an MS RAW file or a channel included in a given RAW file in the case of labeling-based proteomics). Each column corresponds to an attribute/property of the sample or the file, and the value in each cell is the specific value of the property (Fig. 1a).

All the properties in the SDRF must be encoded as ontology terms, whereas the values of the properties can be encoded as ontology terms, numerical values, or free text. To facilitate the annotation, validation, and processing of SDRF files, a list of ontologies has been defined that can be used for encoding each property. For example, most of the sample properties are included in the Experimental Factor Ontology^22^ (EFO—https://www.ebi.ac.uk/efo/), while most of the data-file properties are included in PSI-MS-controlled vocabulary (https://www.ebi.ac.uk/ols/ontologies/ms) and the PRIDE ontology (https://www.ebi.ac.uk/ols/ontologies/pride).

Each sample in an SDRF file has a unique identifier (source name), and every sample property is encoded using the prefix *characteristics* (e.g., *characteristics [organism part]*). Each data file also has a unique identifier (assay name), and every file property has the prefix *comment* (e.g., *comment[instrument], comment[fraction identifier]*). Finally, the variables under study must be specified with the prefix *factor value* (*e.g., factor value[tissue]*). The MAGE-TAB-Proteomics specification defines the minimum information that should be provided for every sample and data file (https://github.com/bigbio/proteomics-metadata-standard/raw/master/psi-document/HUPO-PSI-MAGETAB-Proteomics_latest.docx). For all proteomics experiments, the following properties must be provided: organism, organism part, and biological replicate accession. For every data file, the following properties are required: fraction identifier, technical replicate accession, label (in the case of labeling methods), and data-file name. Biological and technical replicates should be explicitly included using the terms *characteristics[biological replicate]* and *comment[technical replicate]*, respectively (Fig. 1a). The biological replicate field is considered a property of the samples, whereas the technical replicate is considered a property of the data files.

A second category of fields includes information that is not mandatory but recommended. Each PX repository can define which of the recommended fields must be provided in their resource, depending on the experiment types. The current PX templates request the submitters to provide the following properties for every data file: instrument model, cleavage agent, fragment-mass tolerance, precursor-mass tolerance, and mass modifications (e.g., post-translational modifications (PTMs) and artifactual modifications considered in the analysis). Most of the values of these properties can be encoded as a combination of multiple key/value pairs (e.g., methionine oxidation can be specified as AC = UNIMOD:35;NT = Oxidation;MT = Variable;TA = M). We believe that this represents the minimum set of information that is necessary for practical terms for understanding and re-using MS-based proteomics datasets. Furthermore, other properties such as labeling can be provided. Importantly, the set of mandatory properties can be readily expanded in case the PX community decides to extend its metadata requirements in the future. For now, we have defined additional templates (Supplementary Table 1), which form a set of recommended properties required per experiment type. Submitters can use the template corresponding to their experiment to streamline annotation. For example, the cell line (*characteristics[cell line]*) is a recommended metadata item for cell-line experiments; for every human dataset, the disease under study should be provided in the field *characteristics[disease]* and the control samples should be labeled with the value “normal”.

### Multiplexing and fractionation

**Fig. 2:**
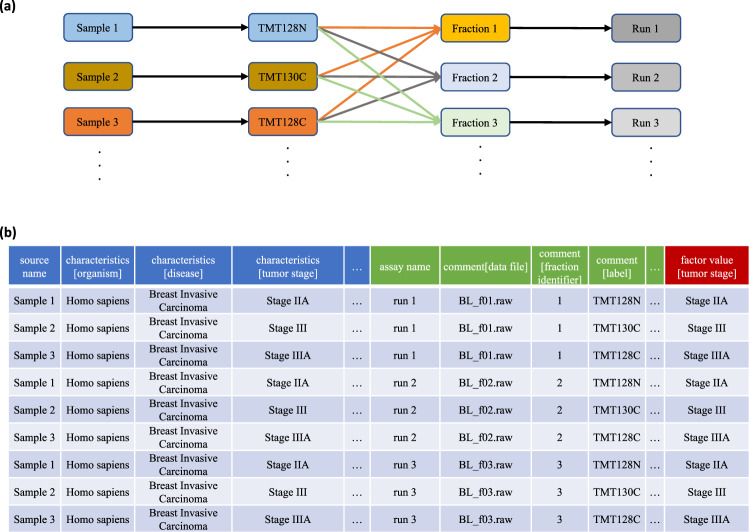
SDRF-Proteomics file for an experiment combining TMT labeling and sample fractionation. **a** TMT experimental design with three samples and three fractions. **b** SDRF representation for a TMT experiment with three samples and three fractions resulting in nine rows where samples are repeated for each fraction and data-file information is repeated for each labeling channel, which is encoded using the property *comment[label]*.

Transcriptomics datasets typically show a one-to-one relationship between each sample and data file. While this is also the case for some proteomics data, two popular experimental designs in proteomics—sample multiplexing and fractionation—follow different patterns (Fig. 2).

In multiplexed quantitative experiments (e.g., based on tandem mass tag (TMT) labeling), multiple samples can be related to the same data file (Fig. 2a). In these cases, the data-file properties should be repeated for each sample including all the properties (e.g., instrument). The different samples included in the same data file can be encoded using the relevant property labels (e.g., comment[label] = TMT128N).

When fractionation is used, the sample information should be repeated for each data file. A property called fraction identifier is used to make clear which fractions correspond to a given data file (e.g., *comment[fraction identifier]* = 1).

These features make MAGE-TAB-Proteomics highly flexible and applicable to complex experimental designs involving both fractionation and multiplexing (Fig. 2b). While the duplication of information can be perceived as redundant, it enables a streamlined data reanalysis because each row/line of the SDRF can be processed individually. In addition, it facilitates meta-analysis with simple operations such as merging different SDRFs coming from different datasets or splitting an SDRF for a given dataset by a specific property of the data file or the sample.

## Crowdsourcing annotations of public proteomics datasets

Crowdsourcing has been successfully applied to address key problems in bioinformatics^23^. The European Bioinformatics Community for Mass Spectrometry (EuBIC-MS^24^, https://eubic-ms.org) set up a collective effort to annotate existing PX datasets using MAGE-TAB-Proteomics^14^. Volunteers from multiple institutes joined the task of annotating, discussing, and improving the SDRF format, openly and collaboratively. Each annotator created an issue in GitHub about a project of interest, forked the main project repository (https://github.com/bigbio/proteomics-metadata-standard), and annotated the corresponding dataset locally in their computers. Then, a pull request was submitted to include the added annotations. Prior to approval, an independent group of reviewers checked that the proposed SDRF conformed to the guidelines. This collaborative peer-review system allowed the identification of potential issues in the file format, which is now compatible with the main MS experiment types. Additionally, a file validator was developed to automatically perform a semantic and structural validation of created SDRF files (see next section for details).

**Fig. 3:**
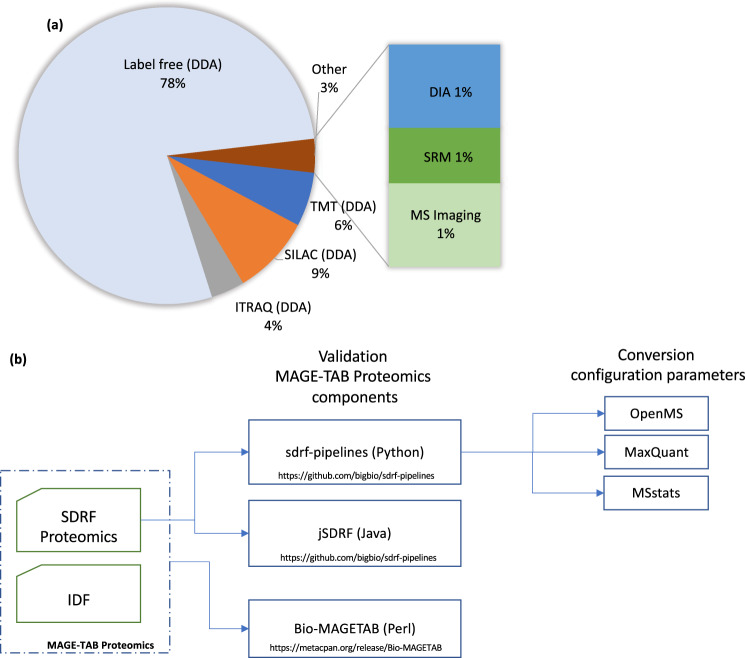
Using MAGE-TAB-Proteomics for dataset annotations. **a** Quantification and data-acquisition methods used in the public datasets that have been annotated with MAGE-Tab-Proteomics by July 2021. **b** Tools and libraries for the validation and conversion of MAGE-TAB-Proteomics files.

As of July 2021, over 200 public datasets have been annotated, covering a broad spectrum of organisms, enrichment/fractionation strategies, quantification approaches, and data-acquisition methods (Fig. 3a). For most multiplexed experiments available on PX, the sample-to-label assignment was not specified by the authors since it was not possible to map the channel to the associated sample in a standardized format. The lack of such essential experimental design information precludes the reprocessing and reproduction of quantitative results. As a consequence, such datasets are underrepresented in the collection of annotated datasets. This highlights the urgent need for systematic and standardized metadata annotations. To support future annotation efforts, Table 1 shows gold-standard annotated datasets from PX that can be utilized as examples when creating an SDRF file for various experimental designs (e.g., label-free quantification, multiplex TMT, stable isotope labeling by amino acids in cell culture (SILAC), affinity purification–mass spectrometry (AP–MS), data-independent acquisition (DIA), and phosphopeptide enrichment).

**Table 1:** Examples of MAGE-TAB-Proteomics files.

Dataset type	Accession code/hyperlink	MAGE-TAB
Label-free	PXD008934	https://github.com/bigbio/proteomics-metadata-standard/tree/master/annotated-projects/PXD008934
TMT, CPTAC dataset not in PX	https://cptac-data-portal.georgetown.edu/study-summary/S029^36^	https://github.com/bigbio/proteomics-metadata-standard/tree/master/annotated-projects/PMID33212010
SILAC	PXD006877	https://github.com/bigbio/proteomics-metadata-standard/tree/master/annotated-projects/PXD006877
Phospho-proteomics	PXD006482	https://github.com/bigbio/proteomics-metadata-standard/tree/master/annotated-projects/PXD006482
Label-free, multiple fragmentation modes and various enzymes	PXD010154	https://github.com/bigbio/proteomics-metadata-standard/tree/master/annotated-projects/PXD010154
AP-MS interactomics	PXD018117	https://github.com/bigbio/proteomics-metadata-standard/tree/master/annotated-projects/PXD018117
TMT	PXD017710	https://github.com/bigbio/proteomics-metadata-standard/tree/master/annotated-projects/PXD017710
Label-free	PXD004242	https://github.com/bigbio/proteomics-metadata-standard/tree/master/annotated-projects/PXD004242
DIA	PXD003539	https://github.com/bigbio/proteomics-metadata-standard/tree/master/annotated-projects/PXD003539
Metabolomics	MSV000086206 [https://doi.org/doi:10.25345/C5HV0S]	https://github.com/bigbio/proteomics-metadata-standard/tree/master/annotated-projects/MSV000086206

## The MAGE-TAB-proteomics toolbox

To facilitate automatic data analysis and reuse, we developed a set of software libraries that enable the validation and conversion from MAGE-TAB-Proteomics format to parameter files (Fig. 3b). For transcriptomics data, the main library to validate a MAGE-TAB file is the Bio-MAGETAB Perl package (https://metacpan.org/release/Bio-MAGETAB). In principle, Bio-MAGETAB can also be used to validate MAGE-TAB-Proteomics files because they are valid MAGE-TAB files. However, Bio-MAGETAB cannot perform extra validations specific to proteomics data, especially the SDRF-Proteomics rules. To enable the validation of the SDRF-Proteomics of MAGE-TAB-Proteomics files, we implemented two libraries in Java and Python (see below). After validation, parameters such as cleavage agents, post-translational modifications, and mass tolerances (precursor and fragment) are translated into MaxQuant and OpenMS parameters in their corresponding configuration files.

The newly developed Python package, called sdrf-pipelines (https://github.com/bigbio/sdrf-pipelines), enables the validation of the structure and also of the semantic rules applied to SDRF. The sdrf-pipelines package can be installed from different package managers like BioConda^25^ or BioContainers^26^. It validates the files according to the different experiments and data types, as defined in the templates. For example, if the template corresponds to a human dataset, the software validates that the sample metadata comply with the human template (organism, disease, ancestry, etc.). In addition, sdrf-pipelines allows users to convert SDRF files to configuration/input files of other popular proteomics analysis tools such as MaxQuant^27^, OpenMS^28^, and MSstats^29^, to facilitate the automation of dataset reanalyses (https://github.com/bigbio/proteomics-metadata-standard/wiki).

The jSDRF Java library (https://github.com/bigbio/jsdrf) we developed also enables the validation of SDRF files. For example, the library can validate that the SDRF contains each sample and data-file relationship, labeling information, fraction identifiers, and the sample and data accessions. It also includes a generic data model that can be used by Java applications to validate and handle SDRF-Proteomics files.

While the PX resources have not yet developed any tool to create SDRF-Proteomics files, existing tools for transcriptomics MAGE-TAB and ISA-TAB can be used to facilitate the process. At the moment of writing, we recommend OntoMaton^30^ (https://github.com/bigbio/proteomics-metadata-standard/wiki/Annotating-Sample-terms-using-OntoMaton), which allows the annotation of Google spreadsheets using ontology terms coming from the Ontology Lookup Service (OLS)^31^. Submitters can search ontology terms in OLS and add them to the sample-property columns. The Google spreadsheets provide the functionalities to copy ontology terms across samples, and to add or remove columns and samples. New versions of the tool will include the possibility to add protein modifications, online validation, and loading existing templates among others.

## Submitting annotated proteomics datasets to the PRIDE database

**Fig. 4:**
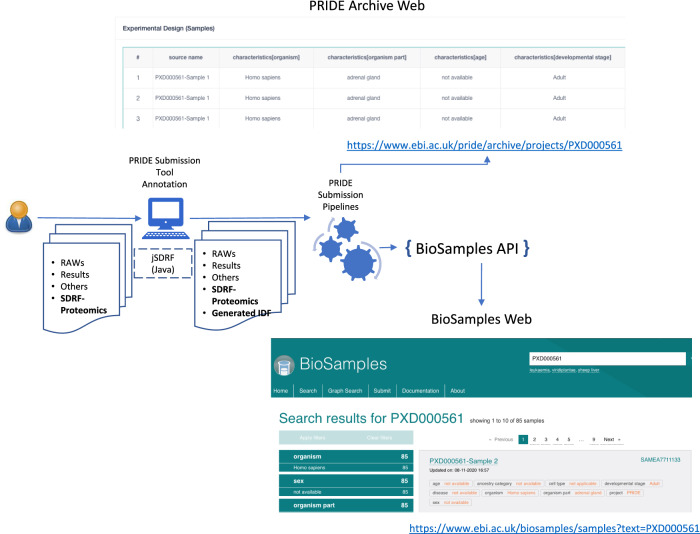
PRIDE database-submission workflow supporting IDF and SDRF files. The IDF file is automatically generated during submission; the SDRF file can be provided by the user in the PRIDE Submission Tool and is automatically validated in the submission pipeline. The sample information is shown on the web page of each PRIDE dataset and submitted to the EBI BioSample database, which assigns a unique accession to each sample. Shown are representative PRIDE and BioSamples outputs for the dataset PXD000561.

Datasets are standardly submitted to PRIDE using the PX submission tool, a desktop application that guides the users through a set of steps to construct the submission and finally performs the transfer of the data files^2^ (Fig. 4). The information annotated by the submitter is encoded into a *submission.px* file. As described above, this PX XML file is highly similar to the MAGE-TAB-Proteomics IDF file. Therefore, the PX XML file is now automatically converted to IDF using PRIDE internal pipelines, once the submission is received and processed.

Since May 2021, the PX submission tool accepts SDRF files. The SDRF file can be created externally as described in the previous section and submitted with the dataset. It is then recognized as an ‘EXPERIMENT DESIGN’ file type and validated using the jSDRF library (Fig. 4). The PRIDE internal pipelines create MAGE-TAB-Proteomics files containing the automatically generated IDF and the user-provided SDRF. Then, the metadata assigned to each sample is automatically submitted to the BioSamples database^32^. A BioSample accession number is created for each sample in the experiment to enable the linking between samples included in multi-omics datasets. The PRIDE web interface presents for each dataset the associated sample metadata table. In addition, the sample metadata are indexed by PRIDE, allowing users to locate and link samples and experiments within the vast number of public datasets.

## Conclusions and future perspectives

Resources such as MassIVE.Quant, ExpressionAtlas, and ProteomicsDB have recently started to systematically reanalyze proteomics public quantitative datasets^4,11,19^. However, the lack of sample metadata that allow associating sample properties with data files makes this task complex and unscalable. Capturing metadata is challenging, since this generic term ranges from concepts such as the title of the experiment to sample-related information, to technical details like the instrument-configuration parameters used for data acquisition. To represent these different levels of proteomics metadata, MAGE-TAB-Proteomics builds upon a popular and flexible data format adopted from transcriptomics that relies on IDF and SDRF files. The IDF-Proteomics format closely resembles the PX XML format that PX resources have long been used to capture dataset information. The SDRF-Proteomics format has a schema and data model that allows submitters to provide the minimum information about the samples as well as a more complete record of their sample and RAW file metadata. The SDRF templates define the minimum information PX partners have agreed upon, which should be provided for each submitted dataset. Additionally, the file format enables users and submitters to describe in much higher detail the sample, sample-processing steps, and the RAW files. Owing to this flexibility, MAGE-TAB is compatible with a wide range of experimental approaches (Table 1) and can be easily adapted to changing metadata requirements in the future. Importantly, the MAGE-TAB-Proteomics project is supported by the world-leading proteomics databases in PX. Due to the gradual and iterative implementation of MAGE-TAB-Proteomics into PX submission pipelines and related tools, the adoption of the standard will not create a major additional burden for users. To support new users, we have created a tutorial in GitHub (https://github.com/bigbio/proteomics-metadata-standard/wiki) and a video that introduces the file format (https://www.youtube.com/watch?v=TMDu_yTzYQM).

MAGE-TAB-Proteomics will facilitate the integration and annotation of proteomics studies, thereby enhancing their discoverability, reproducibility, and reusability. It holds great potential to facilitate the use of standardized workflows for the automated reanalysis of currently available and future annotated public proteomics datasets. This will increase the inherent value of proteomics datasets by making them more amenable for large-scale meta-analysis, systems biology, and multiomics projects that can take advantage of large hardware resources in cloud-based environments. The proposed standard metadata representation will also facilitate the development of common submission systems to streamline the deposition of multiomics datasets to resources for different types of omics data. It may even foster the creation of new multiomics resources, enabling submission, validation, and visualization of multi-omics data in one place^33,34^. As an initial step in this direction, the PRIDE group has started to reanalyze proteomics datasets represented in SDRF and integrates them into the multiomics resource Expression Atlas (e.g., https://www.ebi.ac.uk/gxa/experiments/E-PROT-18/Results).

Expanding the MAGE-TAB-Proteomics format to additional use cases and more types of MS-based experiments, including metaproteomics and xenograft proteomics experimental designs, is currently under discussion (https://github.com/bigbio/proteomics-metadata-standard/blob/master/sdrf-proteomics/use-cases-under-development.adoc). Researchers interested in use cases that are currently not supported are encouraged to bring them to our attention by creating an issue in GitHub using the above hyperlink. Analogous to the implementation process of other PSI formats, we aim to engage software tools and developers to support the MAGE-TAB-Proteomics format as an input file for data processing and as output file for the dataset-submission process. Notably, the MAGE-TAB-Proteomics format has already triggered interest in the MS metabolomics field. First-metabolomics data have already been annotated using MAGE-TAB-Proteomics (Table 1) and more metabolomics datasets coming from the ReDU resource^35^ have been added to the annotation repository, underscoring the open, collaborative, and community-driven approach of this project. In this spirit, we invite all interested parties to join the MAGE-TAB-Proteomics initiative.

## Supplementary information

Supplementary Information

## Data Availability

The annotated datasets generated in this study are provided in GitHub (https://github.com/bigbio/proteomics-metadata-standard/tree/master/annotated-projects). The raw data corresponding to the example datasets shown in Table 1 are available under the accession codes PXD008934, PXD006877, PXD006482, PXD010154, PXD018117, PXD017710, PXD004242, PXD003539, MSV000086206 [https://doi.org/doi:10.25345/C5HV0S]. The raw data of the TMT/CPTAC dataset are available at https://cptac-data-portal.georgetown.edu/study-summary/S029.

## Code availability

General documentation and tutorials as well as a script to generate IDF files are provided in Github (https://github.com/bigbio/proteomics-metadata-standard); however, please note that IDF files are also automatically generated during the PRIDE submission process. The code of the sdrf-pipelines tool developed in this study is deposited in GitHub (https://github.com/bigbio/sdrf-pipelines). The jSDRF Java library is available at https://github.com/bigbio/jsdrf. The Bio-MAGETAB Perl package is available at https://metacpan.org/release/Bio-MAGETAB.
