# Supplementary material for: A proteomics sample metadata representation for multiomics integration and big data analysis
Source: Nat Commun. 2021 Oct 6;12:5854. doi: 10.1038/s41467-021-26111-3 (PMC8494749; doi:10.1038/s41467-021-26111-3)
Supplement: Supplementary file 1 — Supplementary Information [file 41467_2021_26111_MOESM1_ESM.pdf]

A proteomics sample metadata representation for multiomics integration and big data analysis

Chengxin Dai [1], Anja Füllgrabe [2], Julianus Pfeuffer [3], Elizaveta Solovyeva [4,5], Jingwen Deng [1], Pablo Moreno [2], Selvakumar Kamatchinathan [2], Deepti Jaiswal Kundu [2], Nancy George [2], Silvie Fexova [2], Björn Grüning [6], Melanie Christine Föll [7,8], Johannes Griss [9], Marc Vaudel [10], Enrique Audain [11], Marie Locard-Paulet [12], Michael Turewicz [13,14], Martin Eisenacher [13,14], Julian Uszkoreit [13,14], Tim Van Den Bossche [15,16], Veit Schwämmle [17], Henry Webel [12], Stefan Schulze [18], David Bouyssié [19], Savita Jayaram [20], Vinay Kumar Duggineni [20], Patroklos Samaras [21], Mathias Wilhelm [21], Meena Choi [22], Mingxun Wang [23], Oliver Kohlbacher [25,26,,28], Alvis Brazma [2], Irene Papatheodorou [2], Nuno Bandeira [23, 29], Eric W. Deutsch [30], Juan Antonio Vizcaíno [2], Mingze Bai [1, 31,*], Timo Sachsenberg [22,*], Lev Levitsky [5,*], Yasset Perez-Riverol [2,*]

[1] Chongqing Key Laboratory on Big Data for Bio Intelligence, Chongqing University of Posts and telecommunications, Chongqing, China.

[2] European Molecular Biology Laboratory, European Bioinformatics Institute, Wellcome Genome Campus, Hinxton, UK

[3] Algorithmic Bioinformatics, Freie Universität Berlin, Berlin, Germany; Visualization and Data analysis, Zuse Institute Berlin, Berlin, Germany

[4] Moscow Institute of Physics and Technology, Dolgoprudny, Moscow Region, Russia

[5] V.L. Talrose Institute for Energy Problems of Chemical Physics, N.N. Semenov Federal Research Center for Chemical Physics, Russian Academy of Sciences, Moscow, Russia

[6] Bioinformatics Group Department of Computer Science, Albert-Ludwigs-University Freiburg, Freiburg, Germany

[7] Institute for Surgical Pathology, Medical Center – University of Freiburg, Faculty of Medicine, University of Freiburg, Germany

[8] Khoury College of Computer Sciences, Northeastern University, Boston, MA, USA

[9] Department of Dermatology, Medical University of Vienna, Austria

[10] Department of Clinical Sciences, University of Bergen, Norway

[11] Department of Congenital Heart Disease and Pediatric Cardiology, Universitätsklinikum Schleswig-Holstein Kiel, Kiel, Germany

[12] Novo Nordisk Foundation Center for Protein Research, University of Copenhagen, Denmark

[13] Ruhr University Bochum, Medical Faculty, Medizinisches Proteom-Center, 44801 Bochum, German

[14] Ruhr University Bochum, Center for Protein Diagnostics (PRODI), Medical Proteome Analysis, 44801 Bochum, German.

[15] VIB – UGent Center for Medical Biotechnology, VIB, Ghent, Belgium

[16] Department of Biomolecular Medicine, Faculty of Medicine and Health Sciences, Ghent University, Ghent, Belgium.

[17] Department of Biochemistry and Molecular Biology, University of Southern Denmark, Campusvej 55, 5230 Odense, Denmark.

[18] University of Pennsylvania, Department of Biology, Philadelphia, PA 19104, USA

[19] Institute of Pharmacology and Structural Biology, University of Toulouse, CNRS, UPS, France

[20] nference Labs, Bengaluru, KA, 560017, India

[21] Chair of Proteomics and Bioanalytics, Technical University of Munich, Germany

[22] Department of Microchemistry, Proteomics and Lipidomics, Genentech, South San Francisco, CA, USA

[23] Skaggs School of Pharmacy and Pharmaceutical Sciences, University of California San Diego, La Jolla, CA 92093, USA

[25] Institute for Bioinformatics and Medical Informatics, University of Tübingen, Sand 14, 72076, Tübingen, Germany

[26] Department of Computer Science, University of Tübingen, WSI/ZBIT, Sand 14, 72076, Tübingen, Germany

[28] Institute for Translational Bioinformatics, University Hospital Tübingen, Sand 14, 72076, Tübingen, Germany

[29] Center for Computational Mass Spectrometry, Department of Computer Science and Engineering, Skaggs School of Pharmacy and Pharmaceutical Sciences, University of California, San Diego, 92093-0404, USA

[30] Institute for Systems Biology, 401 Terry Ave N, Seattle, WA, 98109, USA

[31] State Key Laboratory of Proteomics, Beijing Proteome Research Center, National Center for Protein Sciences (Beijing), Beijing Institute of Life Omics, Beijing 102206, China

* Corresponding authors: Yasset Perez-Riverol (yperez@ebi.ac.uk), Lev Levitsky (lev.levitsky@phystech.edu), Timo Sachsenberg (sachsenb@informatik.uni-tuebingen.de), Mingze Bai (baimz@cqupt.edu.cn)

Supplementary Note 1: Investigation description format

The IDF (Investigation Description Format) file contains fields describing the study, authors/submitters, protocols, publications (Read Section). ProteomeXchange (PX) resources developed a file format called PX XML (http://proteomecentral.proteomexchange.org/schemas/proteomeXchange-1.4.0.html), which captures the same information as the MAGE-TAB IDF. We have developed a simple Python tool that converts each submission in PRIDE to IDF (https://github.com/bigbio/proteomics-metadata-standard/blob/master/generate_idf.py).

MAGE-TAB Version 1.1
Investigation Title Ultra-deep human phosphoproteome reveals different regulatory nature of Tyr and Ser/Thr-based signaling
Experiment Description Regulatory protein phosphorylation controls nearly every normal and pathophysiological signaling system in eukaryotic cells.

Date of Experiment 2014-08-06
Public Release Date 2014-08-06

Protocol Name P-MTAB-Sample-PXD000612 P-MTAB-Data-PXD000612
Protocol Type sample collection protocol data analysis protocol
Protocol Description HeLa S3 cells were subjected to a double thymidine block in combination with nocodazole Raw mass spectrometric data was analyzed in the MaxQuant environment version 1.5.0.0, and employed Andromeda for database search.
Protocol Parameters
Protocol Hardware Q Exactive
Protocol Software
Protocol Contact

Person Last Name Oroshi Mann
Person First Name Mario Matthias
Person Mid Initials
Person Email oroshi@biochem.mpg.de mmann@biochem.mpg.de
Person Phone
Person Fax
Person Affiliation Proteomics Department of Proteomics and Signal Transduction Max Planck Institute of Biochemistry
Person Address
Person Roles submitter principal investigator
Person Roles Term Source REF
Person Roles Term Accession Number

Experimental Factor Name compound enrichment process

SDRF File PXD000612.sdrf.tsv
Comment[SDRF-Proteomics version] 1.1
Comment[TemplateType] proteomics

Comment[ProteomeXchange accession number] PXD000612

Supplementary Figure 1: IDF for ProteomeXchange dataset PXD000612. Example of a submission.px and their corresponding IDF. The IDF component of a MAGE-TAB-Proteomics document consists of a set of unique tags attached to their corresponding values in a simple tab-delimited text format. For example, "Experiment Description" should be followed by a free-text description of the experiment. Most of the following fields can be used with more than one value, so that multiple values (for example multiple experimental factors) can be defined in a single IDF file. In such cases the multiple values should be separated by semicolons (";")

Some of the tags of the IDF are:

Investigation Title: The overall title of the investigation. This tag can only have one value. Corresponding field in proteomeXchange.xml: Title

Experiment Description: A short paragraph describing the experiment as free-text. This tag can only have one value. The text should clearly explain what you did in your experiment - this will help the curation team to check and process your MAGE-TAB document.
Corresponding field in proteomeXchange.xml: Description

Date of Experiment: The date on which the experiment was performed. This tag can only have one value. Some databases like PRIDE provides the Submission data which can be consider as the Date of the Experiment.

Public Release Date: The date on which the experimental data will be/was released. You can ask us to change this later. This tag can only have one value.
Corresponding field in proteomeXchange.xml: announceDate

Person details: The proteomics community captures Person details differently than the IDF MAGE-TAB specification. The Person information is captured in ProteomeXchange as a list of contacts where each contact is a list of CvTerms. The name of the CVterm is the name of the attribute, value of the CvTerm is the value of the attribute. For example, in <cvParam cvRef="MS" accession="MS:1000586" name="contact name" value="Christoph Krisp"/> the name of the CVTerm is the contact name, the value is the name of the person.

In the IDF, a Contact is a Person with different properties, for example:

- Person Last Name: The last name of each person associated with the experiment.
- Person First Name: The first name of each person associated with the experiment.
- Person Email: The email address of each person associated with the experiment.
- Person Affiliation: The organization affiliation for each person associated with the experiment. This tag is mandatory for sequencing submissions.
- Person Roles: The role(s) performed by each person. Typically, these terms should come from the Experimental Factor Ontology. See for example the list of organization role terms. If more than one role is needed per person, the roles should be given as a semicolon (;) delimited list. The roles defined by ProteomeXchange are two: dataset submitter; or lab head
- Person Roles Term Source REF: The source of the Person Roles terms; his must reference one of the Term Source Names defined in the IDF file.
- Person Roles Term Accession Number: The accession number for this term, taken from the indicated Term Source.

Publication Details:
- PubMed ID: The PubMed IDs of the publication(s) associated with this investigation (where available)
- Publication DOI: A Digital Object Identifier (DOI) for each publication (where available).
- Publication Author List: The list of authors associated with each publication.
- Publication Title: The title of each publication.
- Publication Status: A term describing the status of each publication (e.g. submitted, in preparation, published).

- Publication Status Term Source REF: The source of the Publication Status terms; his must reference one of the Term Source Names defined in the IDF file.
- Publication Status Term Accession Number: The accession number for this term, taken from the indicated Term Source.

Sample and Data Protocols: The sample and data protocols in transcriptomics are captured with a low-level detail, while in proteomics is a summary of multiple protocols within two categories: Sample and Data Protocols. For that reason, we recommend writing the sample and data protocols in the following standard

- Sample Protocol: Protocol Name, The names of the protocols used within the MAGE-TAB document. The sample protocol name for PX submissions will be: *P-MTAB-Sample-PXID*. The protocol name will be the combination of Sample and the Submission PX in ProteomeXchange
- Protocol Type: The type of the protocol, taken from a controlled vocabulary. Typically, this term should come from the Experimental Factor Ontology . See for example the list of protocol terms. The protocol type for PX submissions will be: sample collection protocol
- Protocol Description: A free-text description of the protocol. This text is included in a single tab-delimited field. The Protocol Description is the present Sample Description in ProteomeXchange.
- Protocol Parameters: A semicolon-delimited list of parameter names.
- Protocol Hardware: The protocol hardware is the instrument that was used to capture the sample. If multiple instruments are used, they should be separated by (;)
- Data Protocol: The Data protocol is a generic way in proteomics to capture all the metadata about the data analysis steps.
- Protocol Software: The software used by the protocol.

Experimental Factors:

- Experimental Factor Name: A user-defined name for each experimental factor studied by the experiment. These experimental factors represent the variables within the investigation (e.g. growth condition, genotype, organism part). The actual values of these variables will be listed in the SDRF file, in "Factor Value [<factor name>]" columns.
- Experimental Factor Type: A term describing the type of each experimental factor. These terms will usually come from the Experimental Factor Ontology.
- Experimental Factor Term Source REF: The source of the Experimental Factor Type terms; this must reference one of the Term Source Names defined in the IDF file.
- Experimental Factor Term Accession Number: The accession number for this term, taken from the indicated Term Source.

SDRF File: The name(s) of the SDRF file(s) accompanying this IDF file.

Additional Properties:

- ProteomeXchange accession number: Main identifier of a ProteomeXchange dataset.

Supplementary Table 1: SDRF-Proteomics templates for ProteomeXchange repositories. Minimum sample attributes for primary cells coming from different species and cell lines

	Default	Human	Vertebrates	Non-vertebrates	Plants	Cell lines	Supported Ontology/CV URL for values
Source Name	1	1	1	1	1	1	
characteristics[organism]	1	1	1	1	1	1	https://www.ebi.ac.uk/ols/ontologies/ncbitaxon
characteristics[strain/breed]	0	0	0	0	0	0	https://www.ebi.ac.uk/ols/ontologies/efo
characteristics[ecotype/cultivar]	0	0	0	0	0	0	https://www.ebi.ac.uk/ols/ontologies/efo
characteristics[ancestry category]	0	1	0	0	0	0	https://www.ebi.ac.uk/ols/ontologies/efo
characteristics[age]	0	1	0	0	0	0	
characteristics[sex]	0	1	0	0	0	0	https://www.ebi.ac.uk/ols/ontologies/efo
characteristics[disease]	1	1	1	1	0	1	https://www.ebi.ac.uk/ols/ontologies/efo
characteristics[organism part]	1	1	1	1	1	1	https://www.ebi.ac.uk/ols/ontologies/efo
characteristics[cell type]	1	1	1	1	1	1	https://www.ebi.ac.uk/ols/ontologies/efo
characteristics[individual]	0	1	0	0	0	0	
characteristics[cell line]	0	0	0	0	0	1	https://www.ebi.ac.uk/ols/ontologies/efo
characteristics[biological replicate]	1	1	1	1	1	1	
technology type	1	1	1	1	1	1	proteomic profiling by mass spectrometry
comment[data file]	1	1	1	1	1	1	

comment[fraction identifier]	1	1	1	1	1	1	
comment[label]	1	1	1	1	1	1	https://www.ebi.ac.uk/ols/ontologies/ms<br>https://www.ebi.ac.uk/ols/ontologies/pride
comment[cleavage agent details]	1	1	1	1	1	1	https://www.ebi.ac.uk/ols/ontologies/ms
comment[instrument]	1	1	1	1	1	1	https://www.ebi.ac.uk/ols/ontologies/ms
comment[technical replicate]	1	1	1	1	1	1	

1: Required Attributes for each sample type (e.g. human, vertebrates).
0: Optional Attributes
